# Co-Microencapsulated Black Rice Anthocyanins and Lactic Acid Bacteria: Evidence on Powders Profile and In Vitro Digestion

**DOI:** 10.3390/molecules26092579

**Published:** 2021-04-28

**Authors:** Carmen-Alina Bolea, Mihaela Cotârleț, Elena Enachi, Vasilica Barbu, Nicoleta Stănciuc

**Affiliations:** Faculty of Food Science and Engineering, Dunarea de Jos University of Galati, Romania, 111 Domneasca Street, 800201 Galati, Romania; carmen.bolea@ugal.ro (C.-A.B.); mihaela.cotarlet@ugal.ro (M.C.); elena.ionita@ugal.ro (E.E.); vasilica.barbu@ugal.ro (V.B.)

**Keywords:** microencapsulation, anthocyanins, antioxidant activity, encapsulation efficiency, probiotic

## Abstract

Two multi-functional powders, in terms of anthocyanins from black rice (*Oryza sativa* L.) and lactic acid bacteria (*Lactobacillus paracasei, L. casei 431^®^*) were obtained through co-microencapsulation into a biopolymer matrix composed of milk proteins and inulin. Two extracts were obtained using black rice flour as a raw material and hot water and ethanol as solvents. Both powders (called P1 for aqueous extract and P2 for ethanolic extract) proved to be rich sources of valuable bioactives, with microencapsulation efficiency up to 80%, both for anthocyanins and lactic acid bacteria. A higher content of anthocyanins was found in P1, of 102.91 ± 1.83 mg cyanindin-3-*O*-glucoside (C3G)/g dry weight (DW) when compared with only 27.60 ± 17.36 mg C3G/g DW in P2. The morphological analysis revealed the presence of large, thin, and fragile structures, with different sizes. A different pattern of gastric digestion was observed, with a highly protective effect of the matrix in P1 and a maximum decrease in anthocyanins of approximatively 44% in P2. In intestinal juice, the anthocyanins decreased significantly in P2, reaching a maximum of 97% at the end of digestion; whereas in P1, more than 45% from the initial anthocyanins content remained in the microparticles. Overall, the short-term storage stability test revealed a release of bioactive from P2 and a decrease in P1. The viable cells of lactic acid bacteria after 21 days of storage reached 7 log colony forming units (CFU)/g DW.

## 1. Introduction

Due to the high level of nutrients, black rice (*Oryza sativa* L.) is being increasingly appreciated by consumers and researchers. Previous studies provided several insights into the physiological functionality and processing behavior of the various components of black rice, which can help to promote its more efficient consumption, by increasing consumers’ awareness of this healthy product [1]. Various scientific studies have shown that black rice is a well-balanced food due to its remarkable nutritional properties [2], such as fibers, anthocyanins, vitamins B (thiamine, niacin) and E, iron, magnesium, and phosphorus [2]. The black color is given by the high concentrations in anthocyanins, located in the pericarp and aleuronic layer of the rice [3]. The anthocyanin content of black rice is about 26.3%, with cyanidin-3-*O*-glucoside and peonidin-3-*O*-glucoside representing more than 90% of the total concentration [4]. Anthocyanins from black rice can inhibit the formation of free radicals [5]. The high content of biologically active compounds provides several beneficial properties. The pigmented rice contains compounds with antioxidant activity, such as flavones, proanthocyanidins and phenolic acids, contribute to black rice healthy biological profile [6,7]. However, it is also known that anthocyanins are a class of flavonoids, highly reactive [7], and their stability depends largely on several environmental and chemical factors such as pH, metal ions, light exposure and UV, temperature, oxygen, and enzymatic activity [8]. As a result, anthocyanins present low stability during processing and storage; therefore, their direct use in food formulation, especially in aqueous systems, is difficult [9].

Microencapsulation has been proven to be an effective approach to improve the protection and stability of anthocyanins, by masking flavors and preserving flavors, protecting food against nutritional losses or even to add nutrients after processing. The microencapsulation technology is of particular importance, being widely used to ensure the stability of the active ingredient in food, which may otherwise make the functionality undesirable. The basic principles for achieving the desired stability of the product can be managed by controlling the structural design of the microcapsules, from the perspective of using in food formulations. The microcapsules can comprise many different components, of which the most important is the active ingredient and the polymer matrix, ingredients that must be carefully chosen to control the diffusion rate. Therefore, it is important to understand the morphology, physico-chemical compatibility, and the thermodynamics of both selected components [10].

As such, as one of the most used techniques to protect sensitive compounds, microencapsulation is a technology frequently used to incorporate and immobilize biologically active compounds, such as anthocyanins, within or on solid particles (microspheres) or liquid vesicles [11]. This process also helps light- and heat-sensitive molecules to maintain their stability and to improve their shelf-life, being a rapidly evolving technology with highly specialized advantages and low costs. Although in the food industry, there are different methods of microencapsulation, such as spray drying or coacervation, one of the most used techniques is freeze-drying [12,13].

The aim of this study was to develop a multi-approach experimental based selection of microencapsulation of anthocyanins extracted from black rice flour to develop functional composites for the food applications. Therefore, whey proteins were selected as microencapsulating agents, based on their recognized in vivo biological functions, as they had namely positive influences on the cardiovascular, digestive, endocrine, immune, and nervous systems, whereas multiple uses in the food industry are as a functional food ingredient [14]. Additionally, due to their structural particularities, whey proteins are not easily hydrolyzed by digestive enzymes and their bioactivities can be encrypted in their native protein sequences [15], thus favoring the protection of bioactive compounds in the gastrointestinal tract.

Lactic acid bacteria were selected based on various evidence regarding the potential healthy effect of humans. The application of probiotics has gained much attention among food researchers to formulate and design food ingredients with health-promoting effects [16]. When considering using probiotics in the food industry, many challenges should be considered, especially regarding the viability of these microorganisms during processing and product storage [17], low survival rates in the human gastrointestinal tract (GIT), and low permanence in the intestine. Therefore, maintaining probiotic viability is necessary for promoting health benefits, so a variety of techniques have been applied to protect them until they reach the gut [18], such as microencapsulation. In our study, the black rice flour was used to obtain two extracts: an aqueous extract (E1) and an extract obtained by ultrasound assisted extraction with organic solvents (E2). The two extracts were microencapsulated, using whey protein isolate as the basic encapsulating material and inulin as adjuvant, by freeze drying. The resulting powders (coded P1 and P2) were characterized in terms of encapsulation efficiency, phytochemical content (anthocyanins, polyphenols, and flavonoids), antioxidant activity and cells viability. The structure and morphology of the powders were studied using confocal microscopy. An accelerated storage stability test was performed over a period of 21 days to establish the release on the bioactives from the microcapsules’ and cells’ viability. Moreover, the anthocyanins profile released in vitro in the simulated gastric and intestinal juices was also determined.

## 2. Results and Discussion

### 2.1. Bioactives Profile of the Black Rice Flour Extracts

The fourth fraction, obtained by grinding the black rice flour through a sieve with a mesh diameter of 180 mm, was used for the extraction experiments using ethanolic and aqueous solutions, followed by centrifugation and concentration under vacuum. The two extracts were characterized for anthocyanins, polyphenols, flavonoids, and antioxidant activity (Table 1).

A significant different phytochemicals profile can be observed in Table 1, as expected. The ethanolic extract showed a significantly higher content in all the investigated bioactives. However, both extracts showed a significant content in anthocyanins of 174.90 ± 4.66 mg C3G/g DW and 134.18 ± 0.00 mg C3G/g DW in ethanolic and aqueous extract, respectively. Huang et al. [19] extracted the polyphenols from black rice with methanol a reported values of 5.24 ± 0.05 g GAE/kg in the Black Ch’o-Tzu (CT) variety and a value of 7.36 ± 0.53 g GAE/kg in the Black Kuroo-Mochi (KM) variety. Alvesa et al. [20] used ethanol to extract a bioactive from two types of black rice (long grain and wild rice) and suggested values for polyphenols of 1.52 mg GAE/g and 0.030 mg GAE/g, respectively. The comparative analysis of the extract obtained using ultrasound and conventional extractions, according to [21], showed that the ultrasounds significantly increased the total polyphenols content (TPC) (19.78 and 22.32 mg GAE/g) and the monomeric anthocyanins (30.40 and 35.56 mg C3G/L) while the conventional extraction displayed different values (TPC of 7.53 and 7.78 mg GAE/g, 18.75 and 21.82 mg C3G/L) for the black and purple rice bran, respectively. Shao et al. [22] compared different rice varieties and reported TFC values for the red and black rice grains ranging from 162.86 to 415.10 mg EC/100 g, while TAC was genotype-dependent, ranging from 0.058 to 2.54 mg/g in red rice and 0.015 to 1.41 mg/g in black rice.

### 2.2. The Encapsulation Efficiency and Bioactive Profile of the Powders

One of the most important steps when considering the microencapsulation process is the choice of the most suitable biopolymers as the encapsulating materials, because the type of the encapsulation material is decisive for the physico-chemical and morphological properties of the powders. The type of biopolymer can affect the encapsulation efficiency, shelf-life, and the protection degree of the sensitive encapsulated materials [23].

In our study, two powders were obtained, according to the different extracts. The experimental results showed that the microencapsulation efficiency for the anthocyanins in P1 was 83.56 ± 13.73% and 82.58 ± 1.71% in P2. According to Santos et al. [24], most of the encapsulation technologies can provide a microencapsulation efficiency of up to 99.0%. These authors suggested an encapsulation efficiency of 98.67% for the anthocyanin extract from jabuticaba peels by using ionic gelation. Stănciuc et al. [25] used whey protein isolate and two different polysaccharides (acacia gum and pectin) to encapsulate the grapes’ anthocyanins by coacervation and freeze-drying, with an encapsulation efficiency between 94% and 99%. Huang et al. [26] obtained an encapsulation efficiency for the anthocyanins from black rice extract of 99.45 ± 0.24%. In other studies, such as those of Meng et al. [27], Adachi et al. [28], and Kaimainen et al. [29], different values of the microencapsulation efficiency obtained by water/oil/ water emulsion of 94%, 93%, and 89% were obtained.

The bioactive profile of the powders is given in Table 2. A significant difference may be observed when considering the TAC, with values of 27.60 ± 17.36 mg C3G/g DW for P2 and 102.91 ± 1.83 mg C3G/g DW for P1.

Significantly different values were obtained for TPC, with a lower content in P2 of 113.85 ± 1.92 and a higher content of 200.70 ± 8.34 mg GAE/g DW in P1. Regarding the TFC, P2 showed a significant higher value of 50.97 ± 5.70 mg catechin equivalents (CE)/g DW when compared with 39.77 ± 5.37 mg EC/g DW in P1. Both variants showed high values for antioxidant activity of 87.92 ± 0.54% and 82.50 ± 0.29%, respectively. Aprodu et al. [30] suggested values for the total anthocyanins content (TAC) of 2.95 ± 0.12 mg C3G/g DW and 1.76 ± 0.09 mg C3G/ g DW in two variants of microencapsulated powders, while the total flavonoids contents (TFCs) were 0.31 ± 0.001 and 0.40 ± 0.04 mg quercetin equivalents (QE)/g DW. Both variants had a similar TPC of 1.96 ± 0.31 and 1.98 ± 0.2 mg GAE/g DW.

### 2.3. In Vitro Digestion of the Anthocyanin in the Microencapsulated Powders

**Figure 1:**
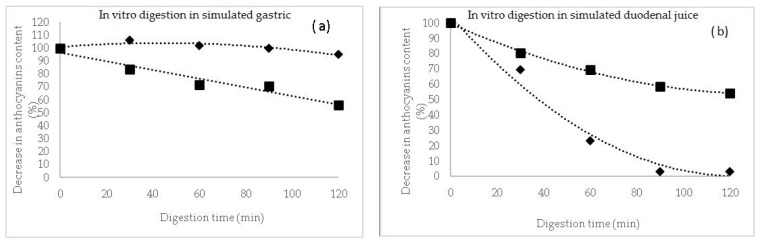
The digestion profile of anthocyanins (%) after in vitro digestion in simulated gastric (**a**) and duodenal juices (**b**) for powders P1 (♦) and P2 (■).

In the case of these microencapsulated powders, a release kinetics of the TAC was performed during the in vitro simulated juices (Figure 1). In the simulated gastric juice (Figure 1a), it can be observed a protective effect of the matrix during the 120 min digestion, for P1, with a slight increase in anthocyanins content in the first 30 min of gastric digestion of approximatively 6%, suggesting a release from the microcapsules. However, during gastric digestion, the maximum decrease in anthocyanins content in P1 reached maximum 5%. A significant different gastric pattern was observed for P2, with a maximum decrease in anthocyanins of approximatively 44% after 120 min of reaction.

Therefore, anthocyanins in P2 were more sensitive to the gastric environment and this might be due to the different profile and chemical structure of the extracted compounds.

In intestinal juice, the anthocyanins decreased significantly in P2, reaching a maximum of 97% at the end of digestion, whereas in P1, more than 45% from the initial anthocyanins content remained in the microparticles (Figure 1b). Flores et al. [31] suggested that a higher concentration of anthocyanins was released from the tescovine ethanolic extract at the end of the gastrointestinal digestion. Furthermore, Kahle et al. [32] reported that a percentage between 28% and 85% of the ingested blueberry polyphenols end up intact in the colon.

Therefore, it can be concluded that the release of the anthocyanin compound is limited in the gastric simulated juice, especially in P1, hence indicating that the whey proteins can be used to protect the anthocyanins in the stomach, allowing a possible release of the bioactive compounds in the gut [33]. It can also be concluded that, in addition to the health benefits of whey proteins, the microencapsulated powder can alleviate the postprandial oxidative stress throughout the gastrointestinal tract [34].

### 2.4. Structure and Morphology of the Microencapsulated Powders

**Figure 2:**
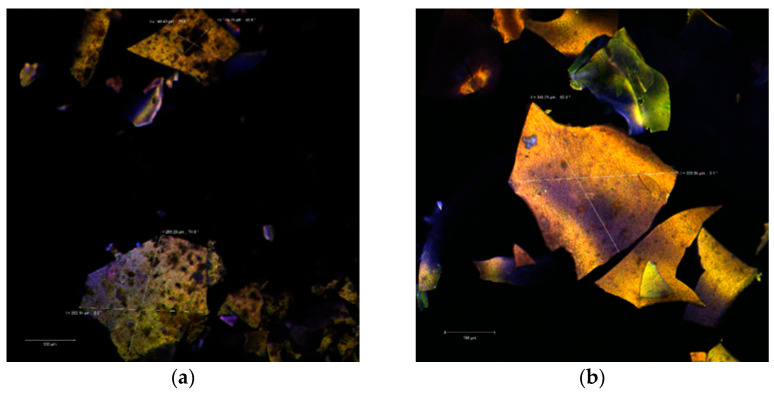

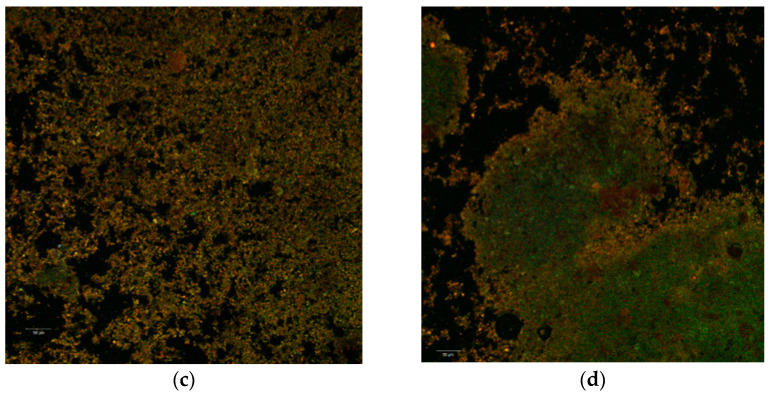
Confocal laser scanning microscopy images of the native microencapsulated powders: Powder II—native state (**a**) and with fluorophore (**c**); Powder I—in native state (**b**) and with fluorophore (**d**).

The confocal analysis did not reveal significant differences between the two powders. In the colorless state, the images captured with the Zen Black software highlighted several irregular polygonal structures. These types of structures were displayed as large, thin, fragile and with the tendency to fracture. The dimensions of these scales were between 128.25–263.25 µm in the case of P2 (Figure 2a) and even larger (339.95–346.21 µm) in the case of P1 (Figure 2b).

Through auto-fluorescence, the numerous phytochemical compounds of the black rice extract emitted in an extremely wide range (550–650 nm) depending on the biochemical profile of the plant source (variety, biotype) or the extraction method. Thus, the biofilm resulted from the microencapsulation of the aqueous extract (P1) is thinner, more homogeneous and with an emission predominantly in yellow. Inside of it, it can be observed the lactic acid bacteria micro-colonies. By staining the powders, the used fluorophore bound to the whey protein isolate (WPI) component and allowed the visualization of the extremely fine biopolymer matrix that trapped the micro-particles (1–2 µm in yellow) of the predominant anthocyanins from the extract as well as the lactic bacteria (in green) as a uniform, homogeneous biofilm.

### 2.5. Storage Stability Test

Both powders were stored at 4 °C for 21 days and characterized in terms of anthocyanins, polyphenols, flavonoids, and antioxidant activity. In Table 3 are given the phytochemical profiles of the powders during storage.

A different pattern may be observed for phytochemical short-term stability in co-microencapsulated powder (Table 3). A slow release of TAC and TPC from P2 may be observed starting with day 7, reaching a maximum of approximatively 5% for anthocyanins and 9% for polyphenols after 21 days of storage. Flavonoids increase reached a maximum value of 28% after 21 days, suggesting a protective effect of the matrix for anthocyanins and polyphenols and less effect on flavonoids. In P1, a decrease in phytochemicals was observed, of approximatively 9% for anthocyanins, 5% for polyphenols and flavonoids. Based on the results, it may be appreciated that both variants of the powders revealed no significant variation of antioxidant activity.

In the study conducted by Amit et al. [35] regarding the microencapsulation of anthocyanins in purple rice, it was illustrated that after the microencapsulation process there may be a significant decrease in the anthocyanin content. A similar type of observation was reported by Weber et al. [36]. Degradation of anthocyanin, during storage, may occur due to various reaction mechanisms, such as condensation reactions, oxidation, and cleavage of a covalent bond. During high-temperature storage, the reaction rate of these reactions is high, which enhance anthocyanin degradation. However, the exact mechanism for anthocyanin degradation during storage is difficult to establish [37].

### 2.6. The Viability of the L. Paracasei within the Microencapsulated Powders

The viability of the *L. paracasei* strain in the co-microencapsulated powders was tested during storage at 4 °C, up to 21 days to test the probiotic properties of the powders. A cell’s viability of 9.89 up to 7.43 log CFU/g DW after 21 days, for P1, which highlighted the highest concentration of the probiotic bacteria, was achieved. For the P2 the cells viability of the co-microencapsulated lactic bacteria with the bioactive compounds from black rice extracts ranged from 9.00 to 6.69 log CFU/g DW, after 21 days.

Enache et al. [38] reported that the co-microencapsulated powder of *L. casei* 431^®^ and the black currant bioactive compounds reached a concentration of probiotics between 8.13 and 6.35 log CFU/g after 90 days of storage, at 4 °C. To describe a functional product, a minimum concentration of probiotics such as 6.00 log CFU/g is required [39].

## 3. Materials and Methods

### 3.1. Black Rice Material

Black rice (*Oryza sativa* L.) was purchased from a local market (Galați, Romania) in January 2020. The black rice was grounded in a laboratory mill, thus obtaining the black rice flour. The obtained flour was sieved through several sieves of different diameters. As previous studies have shown by Bolea et al. [1], the highest content of phytochemicals was identified in fraction four of the seven fractions of black rice flour—a fraction that was obtained through the sieve with the diameter of 180 mm. Therefore, fraction four was further used in the extraction of bioactives, targeting especially anthocyanins.

### 3.2. Reagents and Bacterial Strain

Whey proteins isolate (protein content 95%) was purchased from Fonterra (Auckland, New Zeeland). Inulin, 2,2-Diphenyl-1-picrylhydrazyl (DPPH), 2,2′-azino-bis-3-ethylbenzothiazoline-6-sulfonic acid (ABTS), ethanol, HCl, sodium hydroxide, Folin–Ciocâlteu reagent and gallic acid were obtained from Sigma Aldrich Steinheim, Germany. The probiotic strain of *Lactobacillus paracasei* (*L. casei* 431^®^) was purchased from Chr. Hansen (Hoersholm, Denmark). The viability of the *L. casei* 431^®^ was performed on de Man, Rogosa and Sharpe agar (MRS agar), which was acquired from Merck (Darmstadt, Germany).

### 3.3. The Black Rice Flour Extracts

In the extraction experiments, two types of solvents were used. The first extraction (aqueous extract, further called as E1) was performed from 180 g of black rice with 450 mL of sterile water at 70 °C, the extraction being carried out at room temperature for 24 h, from which 200 mL of extract were taken to be used further for the characterization and microencapsulation experiments.

The second extraction (ethanolic extraction, further called as E2) was performed from 100 g of black rice flour, fraction four, mixed with 400 mL of 70% ethanol solution and 50 mL of HCl 1N. The extraction was performed by stirring for 24 h at room temperature, followed by centrifugation at 5000× *g* for 20 min at 4 °C. The supernatant was concentrated to dryness under reduced pressure at 40 °C. The concentrated extract was weighed and dissolved in 200 mL of ultrapure water, filtered, and further used for the characterization and microencapsulation experiments.

### 3.4. Characterization of Black Rice Flour Extracts

Black rice flour extracts were characterized in terms of total monomeric anthocyanins content (TAC), total polyphenolic content (TPC), total flavonoids content (TFC) and antioxidant activity, as described by Bolea et al. [40]. For the antioxidant activity, the modified ABTS radical discoloration test, was used according to the method described by Miller & Rice-Evans (1997). All the values were expressed as mg/g dry weight of extract (DW). The antioxidant activity was expressed as percentage 2,2-diphenyl-1-picrylhydrazyl free radical scavenging activity (DPPH-RSA) and ABTS.

### 3.5. Co-Microencapsulation of Anthocyanins and Lactic Acid Bacteria

In order to obtain the powders, the same microencapsulation protocol was used, the only difference being the type of the used extract. As such, in the extract solutions, 4 g of whey protein isolate (WPI) and 2 g of inulin were dissolved. The mixtures were allowed to hydrate at 40 °C, for 24 h. The solutions were sterilized by UV radiation, in a microbiological niche (Safe Fast Elite, Milan, Italy) for 2 h, followed by pH adjustment up to 4.5. Finally, 1% (*w/w*) of *L. casei* 431^®^ lyophilized starter culture was inoculated and homogenized on a magnetic stirrer, until its complete dissolution. Then, both solutions were freeze-dried (CHRIST Alpha 1–4 LD plus, Osterode am Harz, Germany) at −42 °C under a pressure of 10 Pa for 48 h, Enache et al. [41]. The obtained powders were stored in sterile brown glass jars, at 4 °C, until further experiments. The resulting powders were coded P1 for the extract obtained by aqueous extraction and P2 for the extract obtained by ethanolic extraction.

### 3.6. The Microencapsulation Efficiency and Powders Phytochemical Profile

The methods described by Oancea et al. [34] were used to evaluate the microencapsulation efficiency and phytochemical profile and the antioxidant activity of powders.

### 3.7. Short-Term Storage Stability Test

The powders were packed in glass jars and stored at 4 °C. The characterization of the biologically active compounds (TAC, TFC, TPC, and antioxidant activity) was determined after 0 and 21 days of storage.

### 3.8. In Vitro Digestion of Anthocyanins from Microcapsules

A static method was used to perform the in vitro digestion of the powders. To simulate the digestion, the powders were firstly mixed with a Tris-HCl buffer (10 mM, pH 7.7), using a ratio of 250 mg powder to 5 mL buffer solution. The simulated gastric fluid (SGF) containing porcine pepsin (20 mg) and 0.1 N HCl (20 mL) at pH 2.0 was added to simulate the stomach conditions. The incubation was performed at 37 °C using an incubator that allows an Optic Ivymen System for orbital agitation (Biotech-SL, Madrid, Spain) at 150× *g*. The anthocyanins content was quantified at 30 min intervals during the in vitro digestion simulation, according to the method described by Stănciuc et al. [25]. Regarding the simulation of the enteric digestion, the simulated intestinal fluid (SIF) consisted of a mixture containing pancreatin (40 mg) and 0.9 M sodium bicarbonate (20 mL), the mixture being adjusted to pH 7.7. The enteric digestion analysis was performed using the simulated intestinal fluid (SIF) juice over which 5 mL of the remaining simulated gastric fluid (SGF) was added after 120 min. The incubation and determination of TAC were performed according to the gastric digestion protocol.

### 3.9. Viability of the Probiotic Strain

The viability assessment was performed for the co-microencapsulated samples before and after the freeze-drying process, until the 21 days of storage, at 4 °C. To count the *L. casei* 431^®^ probiotic culture, the pour plate technique was considered. The viability followed the assessment of the number of colony-forming units by cultivation on the MRS agar, after incubation at 37 °C, for 48 h, under aerobic conditions. The counts were expressed as colony-forming units (CFU) per g DW, Enache et al. [38].

### 3.10. Confocal Laser Scanning Microscopy Analysis

The anthocyanin compounds from the black rice extracts, encapsulated in the whey protein isolate and inulin matrix (WPI-I) by freeze-drying were studied by confocal laser scanning microscopy. To obtain a clear view of the details regarding the particle microstructure, to observe and to determine the structure and morphology of the micro-particles, a LSM 710 Zeiss Confocal Laser Scanning System (Carl Zeiss, Köln, Germany) equipped with a diode laser (405 nm), Ar- laser (458, 488, 514 nm), DPSS laser (diode pumped solid state-561 nm) and HeNe laser (633 nm) was used. To achieve the fluorescent visualization, Congo Red (40 μM) excitation 554 nm/emission 568 nm was used as a fluorophore. The stained samples were observed with a Zeiss Axio Observer Z1 inverted microscope (Carl Zeiss, Köln, Germany) equipped with a 40× apochromatic objective. The obtained images were rendered and analyzed by the ZEN 2012 SP1 software (Black Edition).

### 3.11. Statistical Analysis

All experimental measurements were performed at least in triplicate, and the results are presented as mean value ± standard deviation (SD). The one-way analysis of variance (ANOVA) and Tukey’s test with a 95% confidence interval was applied using Minitab software, version 18, to identify significant differences.

## 4. Conclusions

Two microencapsulated powders containing biologically active compounds from black rice extracts and lactic acid bacteria were obtained in this study, from the perspective to develop products with applicability in different industries, which successfully meet the requests to be used as functional ingredients. The two methods of extraction used revealed a different phytochemical profile of the resulting extracts, with a significantly higher content in bioactives in the ethanolic extract. Significant different values were obtained for TPC, with a lower content in P1 of 113.85 ± 1.92 and a higher content of 200.70 ± 8.34 mg GAE/g DW in P2. Regarding the TFC, P1 showed a significant higher value of 50.97 ± 5.70 mg EC/g DW when compared with 39.77 ± 5.37 mg EC/g DW in P2. A slow release of TAC and TPC from P2 may be observed starting with day 7, reaching a maximum of approximatively 5% for anthocyanins and 9% for polyphenols after 21 days of storage. During the gastric digestion, the maximum decrease in anthocyanins content in P1 reached maximum 5%. A significant different gastric pattern was observed for P2, with a maximum decrease in anthocyanins of approximatively 44% after 120 min of reaction. In intestinal juice, the anthocyanins decreased significantly in P2, reaching a maximum of 97% at the end of digestion, whereas in P1, more than 45% from the initial anthocyanins content remained in the microparticles. The results of this study showed that the microencapsulated black rice extracts are an important source of bioactive compounds that have a high antioxidant activity and can also be used as a growth factor for the *L. casei* 431^®^ probiotic strain.

## Figures and Tables

**Table 1:** Biologically active compounds of the two extracts obtained from black rice flour.

Type of Extract	Total Antochyanins, mg C3G/g DW	Total Polyphenols, mg Gallic Acid Equivalents (GAE)/g DW	Total Flavonoids, mg EC/g DW	2,2’-azino-bis(3-ethylbenzothiazoline-6-sulfonic acid) (ABTS), %
Aqueous extract	134.18 ± 0.02 ^b^	18.43 ± 0.18 ^b^	19.47 ± 0.50 ^b^	75.73 ± 1.28 ^b^
Ethanolic extract	174.90 ± 4.66 ^a^	453.36 ± 6.21 ^a^	428.71 ± 21.32 ^a^	88.92 ± 1.26 ^a^

On each column, means that do not share the same letter (^a,b^) are statistically different at *p* < 0.01 based on the Tukey test.

**Table 2:** Bioactive profile of the powders (P1–the powder that contains the extract obtained by aqueous extraction; P2–the powder that contains the extract obtained by ethanolic extraction).

Powders	Total Anthocyanins, mg C3G/g DW	Total Polyphenols, mg GAE/g DW	Total Flavonoids, mg Catechin Equivalents (CE)/g DW	ABTS, %
P2	27.60 ± 17.36 ^b^	113.85 ± 1.92 ^b^	50.97 ± 5.70 ^a^	82.50 ± 0.29 ^b^
P1	102.91 ± 1.83 ^a^	200.70 ± 8.34 ^a^	39.77 ± 5.37 ^b^	87.92 ± 0.54 ^a^

Means that do not share a letter (^a,b^) on column are significantly different.

**Table 3:** The storage stability test of the powders (P1-the powder that contains the extract obtained by aqueous extraction; P2-the powder that contains the extract obtained by ethanolic extraction).

**P2**				
	**0**	**7 days**	**14 days**	**21 days**
Total anthocyanins, mg C3G/DW	27.60 ± 9.75 ^a^	27.86 ± 3.83 ^a^	28.38 ± 3.89 ^a^	28.96 ± 3.68 ^a^
Total polyphenols, mg GAE/ DW	113.85 ± 1.92 ^a^	118.40 ± 4.28^a^	123.41 ± 4.60 ^a^	123.73 ± 2.26 ^a^
Total flavonoids, mg EC/g DW	50.97 ± 5.70 ^b^	53.61 ± 2.32 ^ab^	63.34 ± 1.40 ^ab^	65.16 ± 4.80 ^a^
2,2-diphenyl-1-picrylhydrazyl (DPPH) %	73.72 ± 1.39 ^a^	75 ± 4.15 ^a^	75.10 ± 1.57 ^a^	76.11 ± 0.25 ^a^
ABTS %	82.50 ± 0.29 ^b^	84.20 ± 1.10 ^ab^	84.40 ± 0.80 ^ab^	85.17 ± 0.78 ^a^
**P1**				
Total anthocyanins, mg C3G/DW	102.91 ± 1.83 ^b^	119.27 ± 2.10 ^a^	97.64 ± 2.43 ^bc^	94.01 ± 6.94 ^c^
Total polyphenols, mg GAE/ DW	200.70 ± 8.34 ^a^	198.45 ± 8.92 ^a^	193.77 ± 2.3 ^a^	190.91 ± 1.43 ^a^
Total flavonoids, mg EC/g DW	39.77 ± 5.37 ^a^	39.26 ± 8.99 ^a^	38.93 ± 1.13 ^a^	37.79 ± 8.47 ^a^
DPPH %	80.27 ± 0,39 ^a^	80.22 ± 2.56 ^a^	80.11 ± 0.54 ^a^	80.06 ± 0.66 ^a^
ABTS %	87.92 ± 0.54 ^a^	85.46 ± 0.05 ^b^	84.19 ± 1.05 ^b^	84.09 ± 1.88 ^a^

On each column, means that do not share the same letter (^a,b,c^) are statistically different at *p* < 0.01 based on the Tukey test.

## Data Availability

In this section, please provide details regarding where data supporting reported results can be found, including links to publicly archived datasets analyzed or generated during the study. Please refer to suggested Data Availability Statements in section “MDPI Research Data Policies” at https://www.mdpi.com/ethics. You might choose to exclude this statement if the study did not report any data.

## Sample Availability

Samples of the compounds are not available from the authors.
